# Weaning failure of cardiovascular origin: how to suspect, detect and treat—a review of the literature

**DOI:** 10.1186/s13613-019-0481-3

**Published:** 2019-01-09

**Authors:** Christina Routsi, Ioannis Stanopoulos, Stelios Kokkoris, Antonios Sideris, Spyros Zakynthinos

**Affiliations:** 1 First Department of Critical Care, Medical School, National and Kapodistrian University of Athens, “Evangelismos” Hospital, Ipsilantou 45-47, 10676 Athens, Greece; 2 Respiratory Failure Unit, Medical School, “G. Papanikolaou” Hospital, Aristotle University, Thessaloníki, Greece; 3 Department of Cardiology, “Evangelismos” Hospital, Athens, Greece

**Keywords:** Weaning from mechanical ventilation, Weaning-induced cardiovascular dysfunction, Cardiovascular drug therapy, Intensive care, Difficult-to-wean patients

## Abstract

Among the multiple causes of weaning failure from mechanical ventilation, cardiovascular dysfunction is increasingly recognized as a quite frequent cause that can be treated successfully. In this review, we summarize the contemporary evidence of the most important clinical and diagnostic aspects of weaning failure of cardiovascular origin with special focus on treatment. Pathophysiological mechanisms are complex and mainly include increase in right and left ventricular preload and afterload and potentially induce myocardial ischemia. Patients at risk include those with preexisting cardiopulmonary disease either known or suspected. Clinically, cardiovascular etiology as a predominant cause or a contributor to weaning failure, though critical for early diagnosis and intervention, may be difficult to be recognized and distinguished from noncardiac causes suggesting the need of high suspicion. A cardiovascular diagnostic workup including bedside echocardiography, lung ultrasound, electrocardiogram and biomarkers of cardiovascular dysfunction or other adjunct techniques and, in selected cases, right heart catheterization and/or coronary angiography, should be obtained to confirm the diagnosis. Official clinical practice guidelines that address treatment of a confirmed weaning-induced cardiovascular dysfunction do not exist. As the etiologies of weaning-induced cardiovascular dysfunction are diverse, principles of management depend on the individual pathophysiological mechanisms, including preload optimization by fluid removal, guided by B-type natriuretic peptide measurement, nitrates administration in excessive afterload and/or myocardial ischemia, contractility improvement in severe systolic dysfunction as well as other rational treatment in specific indications in order to lead to successful weaning from mechanical ventilation.

## Introduction

Among the many causes and pathophysiological mechanisms that impair weaning from mechanical ventilation [1, 2], the respiratory system failure is considered to be the most common, typically viewed as an imbalance between respiratory load and respiratory muscle capacity [3–5]. Cardiovascular dysfunction as an underlying mechanism of weaning failure, though initially described in 1988 by Lemaire et al. in patients with chronic obstructive pulmonary disease (COPD) and concomitant cardiovascular disease [6] was rather underestimated over the next decade. In 2002, the term “weaning-induced cardiac failure” was introduced [7]. Since then, this condition has become more recognizable, but its true incidence is unknown. In an earlier study, Epstein et al. [8] found that as many as one-third of weaning failures resulted solely or in part from “congestive heart failure.” An even higher prevalence of weaning-induced cardiοvascular dysfunction has been reported from experienced centers where this condition is systematically examined [9–13]. Therefore, this entity is currently deemed to be common, particularly in specific subgroups of mechanically ventilated patients such as those with COPD and/or underlying cardiac disease [6, 11–15].

Despite the significant advances in understanding the pathophysiology of weaning failure of cardiovascular origin, studies demonstrating improved weaning outcomes by appropriate treatment are scarce [6, 16–21]. However, despite the lack of adequate evidence demonstrating benefit, in daily clinical practice ICU clinicians caring for mechanically ventilated patients with difficult weaning of cardiovascular origin, frequently make treatment decisions by using pharmacotherapies mainly adopted from the evidence-based non-ICU cardiology.

We conducted a literature review to summarize earlier and contemporary evidence of the most important clinical and diagnostic aspects of cardiovascular origin weaning failure with special focus on treatment.

## Pathophysiological considerations

Knowledge of the changes in the cardiopulmonary pathophysiology that occur during mechanical ventilation and its withdrawal [22, 23], is a prerequisite for early recognition of weaning failure of cardiovascular origin and optimal patient management. The success of weaning depends on the ability of the respiratory system and cardiac pump to tolerate these changes.

**Fig. 1:**
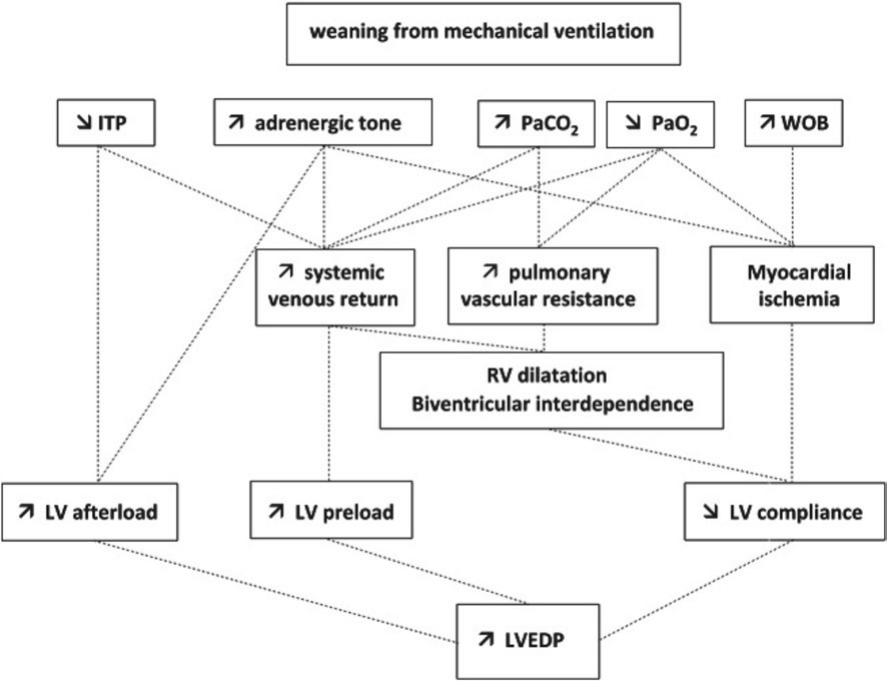
Main mechanisms potentially involved in the development of weaning-induced pulmonary edema. *ITP* intrathoracic pressure, *LV* left ventricular, *LVEDP* left ventricular end-diastolic pressure, *PaO*_2_ oxygen arterial pressure, *PaCO*_2_ carbon dioxide arterial pressure, *RV* right ventricular, *WOB* work of breathing (adapted from [14])

Earlier studies, both experimental and clinical [6, 24–26], give convincing evidence of acute cardiovascular dysfunction as the origin or a cofactor of weaning failure. Briefly, mechanical ventilation with positive airway and intrathoracic pressures reduces venous return and both left ventricular (LV) preload and afterload [22]. On the contrary, the shift from mechanical ventilation to spontaneous breathing induces: first, a negative intrathoracic pressure which, (a) increases the systemic venous return pressure gradient, the right ventricular preload, the central blood volume and the LV preload and (b) increases the surrounding pressure of the LV with a resulting increase in LV afterload; second, an increase in the work of breathing, and, third, an increase in adrenergic tone as documented by an increase in serum catecholamine levels [6, 27]. These main three triggering mechanisms may induce cardiovascular dysfunction clinically expressed by (i) an increase in pulmonary arterial occlusion pressure (PAOP), (ii) increase in LV filling pressure and finally (iii) pulmonary edema. Figure 1, (adapted from [14]), clearly describes the main mechanisms potentially involved in the development of weaning-induced pulmonary edema. These acute hemodynamic effects may have detrimental consequences in patients with cardiovascular disease. Particularly in those patients with coronary artery disease, they may induce localized myocardial ischemia or unmask areas of preexisting marginal function [28]. It is worth noting that mitral regurgitation has been described during failed weaning in the presence of myocardial ischemia [16, 29, 30], further worsening the cardiovascular dysfunction.

Cardiovascular dysfunction during weaning process may involve systolic and/or diastolic LV alterations [11–13, 31–34]. Recent findings emphasize the role of LV diastolic dysfunction with preserved LV contractility as a contributor to weaning failure. Among ICU patients admitted for other reasons than those related to cardiac disease, weaning failure was more frequently due to varying degrees of LV diastolic than systolic dysfunction [33].

## Identification of patients at high risk of weaning failure of cardiovascular origin

Clinically, cardiovascular etiology as a predominant or a contributing factor to weaning failure can be difficult to distinguish from other causes of weaning failure for two reasons: first, this entity may exist at a subclinical level, i.e., some patients may have an almost normal resting cardiovascular function and conditions of increased oxygen demand and consumption, such as a stressful spontaneous breathing trial (SBT), could unmask an underlying cardiovascular dysfunction. Second, both subjective (such as discomfort, dyspnea, anxiety) and objective (tachypnea, tachycardia, wheezing, hypoxemia, hypercapnia) criteria for weaning failure are nonspecific and usually incapable of discriminating cardiac from noncardiac causes of weaning failure [35]. Therefore, weaning failure of cardiovascular origin may be underdiagnosed, and, thus, undertreated due to lack of clinical suspicion.

Classical risk factors for weaning failure of cardiovascular origin mainly include a preexisting cardiopulmonary disease that is either known or suspected [11, 12, 32]. Since, COPD and ischemic heart disease have common risk factors [36, 37], cardiovascular-related weaning failure should be suspected primarily in patients with COPD under mechanical ventilation and presenting difficult weaning. Obviously, the presence of COPD does not identify with certainty a cardiovascular origin of weaning failure. Other causes, e.g., inadequate pharmacological control of the disease, hyperinflation and/or respiratory muscle dysfunction leading to respiratory pump failure must be considered as well.

Silent cardiovascular dysfunction in mechanically ventilated patients with a history of pulmonary disease has been increasingly detected. In a recent study including 107 mechanically ventilated patients with severe exacerbation of COPD, unrecognized previous heart failure was common [38]. Interestingly, the early detection and treatment of heart failure according to current guidelines [39], including administration of diuretics, angiotensin-converting enzyme inhibitors or selective β1-blockers, shortened the length of mechanical ventilation and ICU stay and decreased hospital mortality, indicating the effectiveness of appropriate treatment on the outcome.

In a study of extubation failure [40], a subset of patients with high risk of re-intubation was identified, including those over 65 years old and/or having underlying cardiac or respiratory disease, whereas other factors such as disease severity, mechanical ventilation duration, or arterial blood gases values were not significant. Similarly, previous COPD and “cardiopathy” were factors that were independently associated with weaning-induced pulmonary edema [12]. In another study among nonselected mechanically ventilated patients, Caille et al. [11] observed a high incidence of weaning failure of cardiovascular origin in a subset of patients who failed to wean. Compared to those who succeeded, patients who failed had a significantly lower LV ejection fraction and higher filling pressures before the start of SBT, underlining the contribution of LV systolic dysfunction to weaning failure. Similarly, severe systolic LV dysfunction was an independent factor for extubation failure in a recent study [32]. In summary, in clinical practice, in any unexpected difficulty in weaning, the possibility of an underlying cardiovascular dysfunction should be raised, provided an apparent recovery of adequate respiratory function.

## Diagnostic methods to detect cardiovascular dysfunction in patients with difficult weaning

**Fig. 2:**
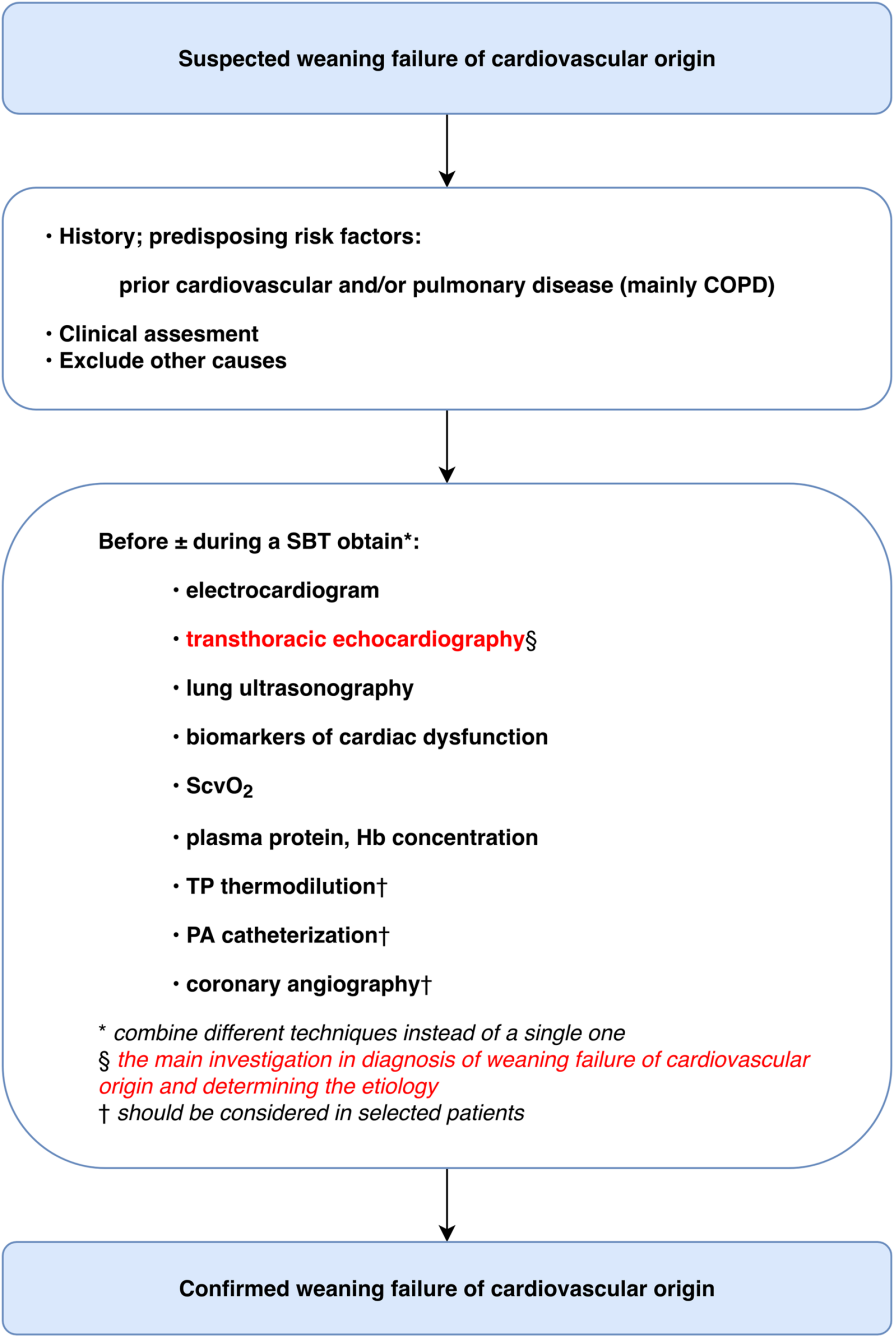
Workup to detect the cardiovascular origin of weaning failure: a general approach. *COPD* chronic obstructive pulmonary disease, *SBT* spontaneous breathing trial, *ScvO*_2_ central venous oxygen saturation, *TP* transpulmonary, *PA* pulmonary artery, *Hb* Hemoglobin

Upon suspicion of cardiovascular origin of weaning failure, a diagnostic workup should be obtained in order to distinguish between cardiac and noncardiac causes of weaning failure. This kind of evaluation is crucial as it may have direct therapeutic implications. The following part constitutes a brief review of the readily available methods that are considered to confirm a cardiovascular-related weaning failure (Fig. 2). These methods can be used either alone or in combination.

**Fig. 3:**
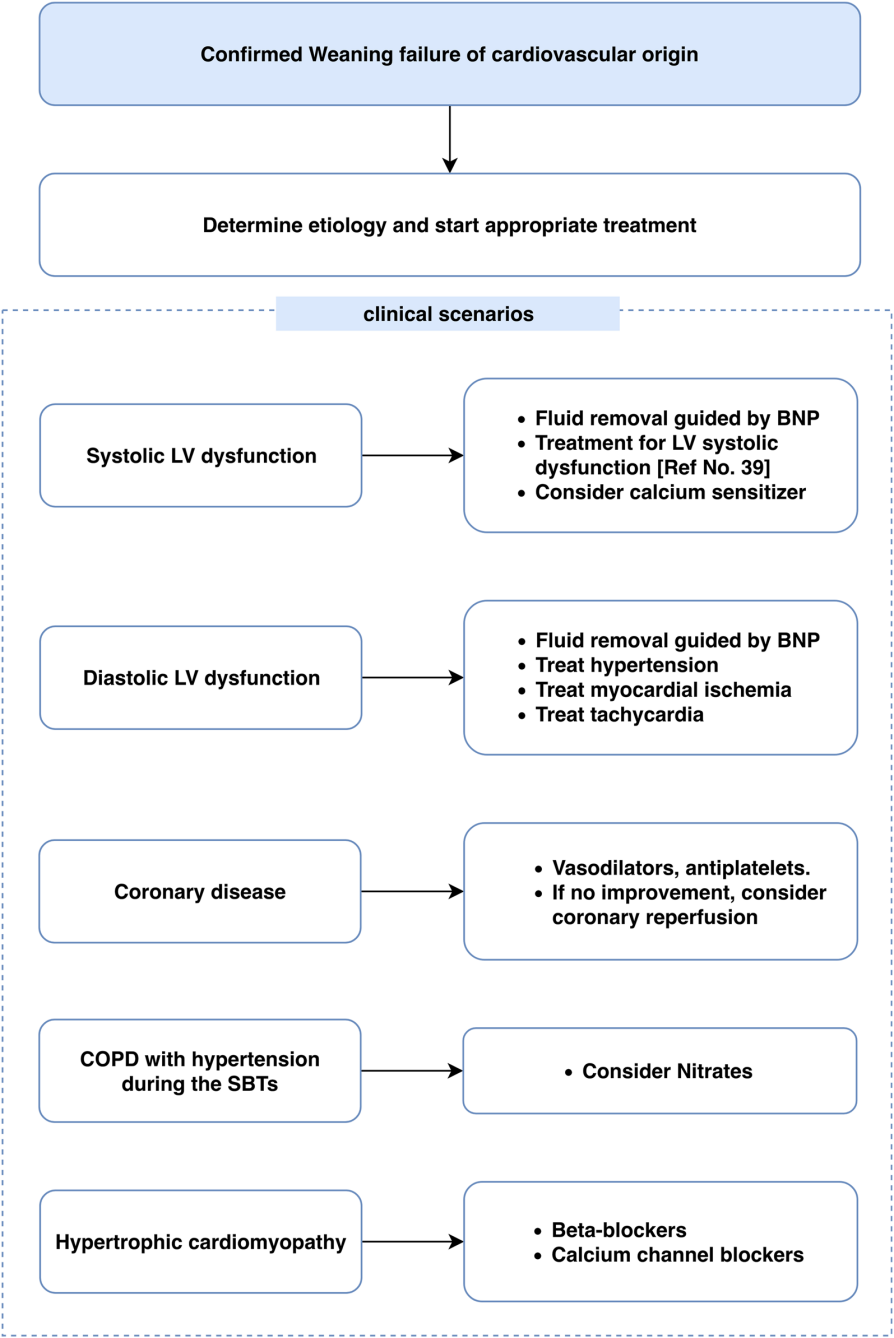
Suggested treatment for weaning failure of cardiovascular origin, according to the etiology. *LV* left ventricular, *BNP* B-type natriuretic peptide, *COPD* chronic obstructive pulmonary disease, *SBT* spontaneous breathing trial, *ACE* angiotensin-converting enzyme

### Pulmonary artery catheterization

Traditionally, weaning-related cardiovascular dysfunction has been defined as the association of signs of clinical intolerance and a PAOP of 18 mmHg or higher during a SBT [6, 10, 41, 42]. Since active expiration due to expiratory muscle contraction is usual during weaning failure, resulting in overestimation of PAOP, the PAOP value should be corrected for this. An esophageal balloon positioning allows pleural pressure measurement to obtain transmural pressure as the difference between PAOP and pleural pressure at expiration [6, 43].

Additionally, pulmonary artery catheter permits hemoglobin oxygen saturation of mixed venous blood (SvO_2_) measurement or continuous recording. In fact, patients detected with an increase in PAOP during SBTs, usually experience a substantial decline in SvO_2_ during SBTs [44, 45] that reflects inadequate oxygen delivery for the increased oxygen consumption, indicating, thus, the contribution of cardiovascular dysfunction. These patients have a high likelihood of failure to wean. In contrast, SvO_2_ usually remains unchanged in patients who are successfully weaned from mechanical ventilation [44]. However, a subset of failed-to-wean patients whose SvO_2_ does not decrease has been described [45]. These patients do not have increased oxygen consumption during weaning failure, possibly due to respiratory center depression in some of the cases.

Today, pulmonary artery catheters are less commonly used, compared to the past, due to lack of evidence concerning the benefits of right heart catheterization [46]. When cardiovascular origin of weaning failure is suspected, a number of non- or less invasive diagnostic methods are now available [47]. These methods are presented in the following paragraphs.

### Critical care echocardiography

Critical care echocardiography combines transthoracic echocardiography and chest ultrasonography examination which have both been increasingly used to identify noninvasively, at bedside the diagnosis and the mechanism of weaning failure of cardiovascular origin [48].

#### Transthoracic echocardiography

In addition to the conventional evaluation of structural and systolic properties of the heart, echocardiography can diagnose diastolic dysfunction by the use of tissue Doppler imaging (TDI). By measurement of the early (E) and late (A) peak diastolic wave velocities at the mitral valve, using pulsed-wave Doppler, and of the early (e′) and late (a′) diastolic wave velocities at the lateral mitral valve annulus by using TDI, echocardiography can reliably demonstrate LV relaxation (e′ wave) and the grade of LV diastolic dysfunction by calculating the E/e′ ratio [49].

In a selected population of difficult-to-wean patients, Lamia and colleagues found that at the end of a SBT, the combination of E/A > 0.95 and E/e′ > 8.5 allowed an accurate detection of weaning-induced PAOP elevation associated with LV diastolic dysfunction [50]. Similarly, in other studies, irrespectively of the presence of diastolic dysfunction at rest, echocardiography, performed before the beginning and over the course of a SBT, explored the evolution of LV contractility and relaxation, detecting thus, cardiovascular dysfunction [11, 13, 33, 51–53]. Remarkably, in some studies, a sole TTE performed before conducting the SBT provided early detection of those patients with impaired LV diastolic function, who were at high risk of a subsequent SBT failure [11, 33, 34].

As latent cardiac failure can become evident during stress, a stress echocardiography (using dobutamine to assess diastolic function as well as ephedrine to evaluate functional mitral regurgitation) was recently used to identify stress-related myocardial dysfunction during the weaning period in patients with resolved cardiogenic shock who experienced weaning failure [54]. It was proved helpful in detecting silent diastolic and systolic dysfunction or severe mitral regurgitation that could have a major impact on weaning outcome.

#### Chest ultrasonography

Lung ultrasound represents a valuable adjunct for evaluating whether respiratory failure is related to cardiogenic pulmonary edema or not [55]. Mongodi et al. [56] described a case of a patient with an unexpected cardiogenic cause of weaning failure to be uncovered by the combined use of transthoracic echocardiography and lung ultrasound, leading, thus, to subsequent appropriate patient management. On the other hand, combined uses of ultrasonography are highly helpful in ruling out a cardiogenic origin and identifying alternative causes of weaning failure, like diaphragmatic dysfunction [57]. Both established and innovative applications of ultrasonography during the weaning process, including assessment of the heart, the diaphragm, the pleura and the lung have been recently described in an excellent review [58].

In summary, bedside critical care echocardiography is a convenient tool to help in the diagnostic workup of weaning failure of cardiovascular origin. Patient-related factors (poor echocardiographic window, particularly among COPD patients, edema, obesity, surgical dressings and significant valvulopathies that can affect the Doppler parameters), equipment quality and clinical skills remain the main limitations in this setting.

### Electrocardiogram

A low incidence of electrocardiogram (ECG) changes indicating ischemia during weaning has been reported in a general ICU population [59–61]. However, this method suffers from a lack of sensitivity. Indeed, LV wall motion abnormalities occurring or worsening during weaning from mechanical ventilation, detected by myocardial perfusion scintigraphy studies, have been documented without concomitant ECG changes [6, 62].

### Biochemical indices of weaning-induced cardiovascular dysfunction

Biomarkers of the volume status and functional state of the heart including serum B-type natriuretic peptide (BNP) and/or N-terminal (NT)-proBNP, have been used as surrogate markers of cardiovascular dysfunction in patients with difficult weaning. High basal levels of BNP or NT-proBNP [34, 42, 63] and/or a BNP increase at SBT completion [10, 15, 34, 42, 64, 65] have been shown to be associated with an increased risk of weaning failure, possibly indicating an underestimated cardiovascular dysfunction. Dres et al., using the pulmonary artery catheter as the reference method, demonstrated that SBT-induced increases in BNP level by more than 12% allowed detection of increases in PAOP and, therefore, the diagnosis of weaning-induced pulmonary edema contributing to weaning failure, with a sensitivity of 76% and a specificity of 78% [10]. Furthermore, a relation between BNP levels and LV diastolic dysfunction grading has been demonstrated in mechanically ventilated patients considered for weaning [34], just like it had been previously shown in patients with heart failure [66]. The short half-life of BNP (22 min) compared with the longer one of NT-proBNP (120 min) [42, 67] suggests that BNP measurement should be more relevant in the context of weaning. Moreover, important to note, BNP could be increased in renal impairment, thus, it should be taken into account for the analysis of the BNP plasma kinetics.

### Blood volume contraction markers

An acute increase in plasma protein or hemoglobin concentration, both greater than 5% during a SBT, reflecting the blood volume contraction induced by pulmonary edema formation has been demonstrated to correlate with the reference diagnostic method, i.e., PAOP increase above 18 mmHg, with acceptable diagnostic power [10, 68]. Thus, it has been proposed as an indirect indication of weaning-induced cardiovascular dysfunction.

### Extravascular lung water

Transpulmonary thermodilution provides direct estimation of pulmonary edema through calculation of the extravascular lung water (EVLW) [69]. The ability of EVLW measurement to detect pulmonary congestion during weaning from mechanical ventilation has been investigated [10]. An increase in indexed EVLW by more than 14% during SBT was able to diagnose weaning-induced pulmonary edema with a sensitivity of 67% and a specificity of 100%. However, transpulmonary thermodilution devices are not widely applied during a weaning process, unless they had been previously used and they are still in place.

### Passive leg raising

Passive leg raising (PLR) by 45 degrees is a dynamic test to assess cardiac preload status and fluid responsiveness. By measuring real-time changes in cardiac output, PLR test is considered to be either positive, if cardiac output increases (indicating preload dependence), or negative, if no augmentation of cardiac output occurs (indicating preload independence) [70]. Based on an expected cardiac preload increase during transmission from mechanical ventilation to spontaneous breathing, PLR has been evaluated to assess the risk of weaning-induced cardiovascular dysfunction. It was found that a negative PLR test prior to SBT indicating preload independence, predicted weaning failure due to cardiovascular dysfunction with a sensitivity of 97% and specificity of 81% [41].

## Treatment strategies in weaning failure of cardiovascular origin

Since cardiovascular dysfunction could be considered as a modifiable factor of weaning failure, appropriate treatment should be started upon confirmation by using one or more of the aforementioned diagnostic methods. Even if the case of cardiovascular dysfunction is not the sole mechanism of weaning failure, it is probably the only one that could be easily addressed and that with respect to the load capacity respiratory balance, improving even slightly one of its determinants might overall improve patient’s outcome.

Large controlled studies systematically investigating the effectiveness of cardiovascular agents and/or other adjuvant treatment to facilitate difficult weaning of cardiovascular origin were not found. However, therapeutic options are available from a limited number of small-sized studies or case studies and case series reports, supporting appropriate treatment to optimize the management of weaning-induced cardiovascular dysfunction. As a result, there are no evidence-based recommendations by international guidelines for the management of weaning failure of cardiovascular origin. However, proposed recommendations by experts including algorithmic approaches have been provided so far [16, 71].

Clinical data on the cardiovascular agents that have been used for the treatment of weaning failure are shown in Table 1.

**Table 1:** Studies with cardiovascular agents that have been used in patients who are difficult-to-wean from mechanical ventilation

Study (References)	No. of patients	Study population diagnosis	Agent (*class*)	Indication given for	Effect	Main findings/comments
Lemaire et al. [6]	15	COPD with concomitant cardiovascular disease	Furosemide (*loop of Henle diuretic*)	Increased preload/fluid management/Hypervolemia	Preload reduction	9/15 patients were successfully weaned
Aubier et al. [81]	8	COPD	Dopamine (*catecholamine*) 10 µg/kg	Impaired diaphragmatic function	Some vasopressor, increase splanchnic blood flow	Results of weaning outcome not reported
Valtier et al. [21]	6	Coronary artery disease	Enoximone (*phosphodiesterase* -*3 inhibitor*)	LV dysfunction	Prevention of weaning-induced LV dysfunction	5/6 patients were successfully weaned
Paulus et al. [20]	9	LV failure after cardiac surgery	Enoximone (*phosphodiesterase* -*3 inhibitor*) 30 µg/kg/min followed by 10 µg/kg/min	LV failure	Increase in cardiac index, no change in PAOP	7/9 patients were successfully weaned
Duane et al. [99]	1	Trauma patient with coronary artery disease	Esmolol IV (*beta*-*blockers*) 500 µg followed by 50–100 µg/kg/min	Tachycardia, hypertension and pulmonary edema upon spontaneous breathing	Normal heart rate and systolic blood pressure	Successfully weaned
Adamopoulos et al. [98]	2	Postoperative patients with hypertrophic obstructive cardiomyopathy	Atenolol 200 mg/day (*beta*-*blockers*) + Diltiazem 300 mg/day (*calcium channel blocker*)	hypertrophic obstructive cardiomyopathy	Decreased dynamic LV obstruction (induced by catecholamines), improved LV compliance	Successfully weaned
Ng et al. [95]	1	Secondary pulmonary hypertension and RV dysfunction	Sildenafil (*Phosphodiesterase*-*5 inhibitor*) 12.5–25 mg 3 times/day	severe pulmonary hypertension	Decrease in PAP and PVR	Successfully weaned from INO and mechanical ventilation
Stanopoulos et al. [19]	3	COPD	Sildenafil (*Phosphodiesterase*-*5 inhibitor*) 50 mg	Pulmonary hypertension	Decrease in PAP and PAOP	Successfully weaned
Sterba et al. [17]	12	Patients with LVEF < 40%	Levosimendan (*Calcium sensitizer*) 0.108–0.21 μg/kg/min, loading dose 12 µg/kg in 6 patients	Impaired LVEF	Increase in LVEF	7/12 successfully weaned
Meaudre et al. [88]	1	Dilatated cardiomyopathy	Levosimendan (*Calcium sensitizer*) loading dose 12 µg/kg followed by 0.1 µg/kg/min × 24 h	Impaired LVEF High LV filling pressures	Increase in LVEF and a decrease in cardiac filling pressures	Successfully weaned
Routsi et al. [18]	12	COPD exhibiting systemic arterial hypertension during SBT	Nitroglycerin (*vasodilator*) 40–600 µg/min	Increased LV afterload	Decrease in LV filling pressures and afterload	10/12 patients successfully weaned
Ouanes-Besbe et al. [83]	10	COPD and normal LVEF	Dobutamine (*beta*-*agonist*) 7µ/kg/min followed by Levosimendan (*Calcium sensitizer*) 0.2µ/kg/min × 24 h	PAOP increase ≥ 10 mmHg during SBT	PAOP and PAP increased to a lesser extent with Levosimendan than with Dobutamine	Successfully weaned Dobutamine increased the rate-pressure product (No indication according to guidelines [39],(see text)
Elias et al. [94]	1	Interstitial lung disease, pulmonary hypertension	Sildenafil (*Phosphodiesterase*-*5 inhibitor*) 20–30 mg^3^ times/day	Severe pulmonary hypertension and a patent foramen ovale	Decrease in PAP	Successfully weaned from INO and mechanical ventilation
Mekontso Dessap et al. [65]^a^	304	General ICU patients eligible for weaning. BNP-driven or physician-driven fluid management	Furosemide (*loop of Henle diuretic*) ± Acetazolamide sodium (*carbonic anhydrase inhibitor*)	Fluid overload	More negative fluid balance in BNP-guided group	Shorter duration of MV in BNP-guided approach. Strongest effect in patients with LV systolic dysfunction
Cateano et al. [89]	1	Aortic stenosis, LV systolic dysfunction	Levosimendan (*Calcium sensitizer*) 0.1 µg/kg/min × 24 h	LV systolic dysfunction	LV systolic function and mean aortic gradient increased	Successfully weaned
Mongodi et al. [56]	1	COPD, arterial hypertension, rheumatoid arthritis	Ramipril (*angiotensin*-*converting enzyme inhibitors*) 2.5 mg/die, Nebivolol (*beta*-*blockers*) 5 mg/die)and mild negative water balance	LV diastolic dysfunction, LUS consistent with increased EVLW	Reduced LV filling pressures and normal LUS	Successfully weaned

^a^Randomized control trial, *COPD* chronic obstructive pulmonary disease, *RV* right ventricular, *LV* left ventricular, *PAOP* pulmonary artery occlusion pressure, *PAP* pulmonary artery pressure, *PVR* pulmonary vascular resistance, *LVEF* left ventricular injection fraction, *RV* right ventricular, *SBT* spontaneous breathing trial, *INO* inhaled nitric oxide, *BNP* B-type natriuretic peptide, *EVLW* extravascular lung water, *LUS* lung ultrasound

### Pharmacological treatments (Fig. 3)

1. Fluid management/Volume overload/Diuretics

Fluid could be considered as a drug that can be overdosed in the ICU. Large volume fluid infusion during initial resuscitation of acute critical illness results in fluid overload and fluid retention, clinically presented as pulmonary and peripheral edema. Negative fluid balance is an expected result of resolution of the pathophysiological process of acute illness and/or the result of an intervention either pharmaceutical, i.e., administration of diuretics, or even renal replacement therapy.

Several studies have shown that fluid overload could be associated with failure to wean from mechanical ventilation [6, 12, 72, 73]. The first randomized study to address fluid management during weaning has been reported by Mekontso Dessap et al. [65]. In mechanically ventilated patients who fulfilled criteria for weaning attempts, the authors showed that fluid management strategy guided by daily BNP plasma concentrations, compared to the physician-guided strategy, decreased the duration of weaning without increasing adverse consequences on hemodynamics or renal function. Compared with the control group, the BNP-guided group had a higher proportion of patients receiving diuretics (furosemide and acetazolamide), resulting thus, in a significantly more negative fluid balance. Particularly, the subgroup of patients with LV systolic dysfunction showed the best-possible beneficial effect of this strategy. Since BNP levels reflect the changes in LV wall stretch and correlate closely with filling pressures in patients with LV dysfunction [66, 74], presumably the negative fluid balance prevented cardiac preload increase, thereby protecting these patients from the development of pulmonary edema during weaning. This explanation is further supported by the finding that in other groups of patients, such as pure COPD patients, such beneficial effect was not found, probably because other pathophysiological causes, such as impaired respiratory mechanics leading to increased respiratory load mainly contributed to weaning difficulties [3–5]. In addition, the benefit of PLR-guided fluid removal guided by a PLR test has been shown [12, 41]. These data actually confirm previous knowledge coming from the landmark study of Lemaire et al. [6], on the development of cardiogenic pulmonary edema during weaning of difficult-to-wean COPD patients with concomitant cardiovascular disease. These patients received intravenous furosemide (80 mg/day) which resulted in a mean body weight reduction of 5 kg within a 10-day period due to fluid loss. As a result, a lower transmural PAOP was measured in eight patients who had undergone a repeated hemodynamic evaluation, and, thereafter, the majority of the patients (9/15) were successfully weaned from mechanical ventilation.

Consequently, since correction of fluid balance is an absolutely modifiable variable, it is of great importance and deserves a routine attention by ICU clinicians. However, overtreatment with diuretics resulting in undesirable side effects including dehydration, hypovolemia and sometimes hypotension and renal dysfunction, is an imminent risk. Particularly, in patients with heart failure and preserved ejection fraction (i.e., LV diastolic dysfunction), aggressive diuresis may result in cardiac output reduction, since these patients are highly sensitive to volume changes and generally have a narrow window between volume overload, causing congestive symptoms and hypovolemia leading to circulatory shock [75]. As a result, recognition of the potential hazards and individualized management is considered to be mandatory in order to protect the patient from inappropriate diuretic dosage. In brief, assessment of the volume and preload status is necessary, as only weaning failure due to excessive preload should be treated with diuretics.

#### Acetazolamide

As a side effect, diuretics may cause metabolic alkalosis which may depress cardiac output and/or central respiratory drive contributing, thus, to weaning failure or delay, especially in COPD patients. Acetazolamide, a carbonic anhydrase inhibitor, has been used to reverse metabolic alkalosis when fluid and potassium replacements are insufficient to correct blood alkalinity. In the aforementioned study by Mekontso Dessap et al. [65], when metabolic alkalosis was present, acetazolamide was added (250 mg every 8 h if pH > 7.45 or 500 mg every 8 h if pH > 7.50) in the absence of contraindications. However, in a randomized controlled trial, among mechanically ventilated COPD patients, the use of acetazolamide did not result in a significant reduction in the duration of mechanical ventilation [76]. It is important to note that the pharmacological effect of acetazolamide lasts longer than expected from the plasma half-life of the drug (5–6 h), as it has been shown in patients with COPD and pure metabolic alkalosis [77]. Therefore, it should be given with caution.

(2) Control of hypertension

Systolic blood pressure of more than 180 mmHg or pressure change greater than 20% during SBT is among the criteria of clinical intolerance and indicators of weaning failure [35]. The etiology of the increased blood pressure during a SBT is most likely multivariable in origin, mainly triggered by hypoxemia, hypercapnia and enhanced sympathetic activity [6, 27]. Additionally, pain, anxiety and withdrawal syndromes, usually from opioid use, are also responsible. Therefore, any antihypertensive therapy should be given only after exclusion of these reversible factors.

Obviously, patients with chronic hypertension should be treated before any weaning attempt. There are a lot of treatment options including beta-blocking agents, calcium channel blockers, vasodilators and angiotensin-converting enzyme inhibitors; the latter providing recent evidence of their benefits in ICU patients with acute kidney injury [78]. These agents decrease arterial pressure via different modes of action, which may be appropriate or contraindicated in individual patients. Selecting the right drug after individualized evaluation is fundamental. As a general rule, pharmaceutical agents with rapid onset and short half-life/duration of action are preferable [79].

In almost all studies of weaning failure of cardiovascular origin, systolic blood pressure increased immediately after the start of an unsuccessful SBT [6, 15, 18, 19, 44]. As it has been shown by Jubran et al. this rise in systolic blood pressure during unsuccessful SBTs was associated with a lack of change in cardiac index, implying an increase in LV afterload [44]. Therefore, optimal therapy should include agents capable of decreasing high arterial blood pressure through reduction in the afterload, such as vasodilators.

In hyperadrenergic states, where tachycardia is typically present, selective beta1-blocking agents are good options for the control of hypertension. Clonidine, a central-acting a2-agonist, may also be a good alternative. It induces a decrease in the sympathetic tone with a significant reduction in blood pressure. It is most useful in patients with drug or alcohol withdrawal and also in those patients in whom a vasodilatory effect is best avoided, e.g., in the presence of intracranial hypertension.

(3) Vasoactive cardiovascular agents

### Catecholamines

#### Dopamine

Dopamine, at infusion rates of 2–15 μg/kg/min, stimulates β1-receptors and increases myocardial contractility as well as splanchnic blood flow at the cost of tachycardia and increased risk of arrhythmias [80]. In an earlier study, Aubier and colleagues [81] investigated the effect of dopamine on diaphragmatic strength and blood flow in COPD patients mechanically ventilated because of acute on chronic respiratory failure. Following a dose of 10 µg/kg during 30 min heart rate increased by 17% and cardiac output by 40% on average, accompanied by a marked increase in diaphragmatic blood flow and transdiaphragmatic pressure. Although the authors demonstrated these favorable effects of dopamine on diaphragmatic function, weaning outcome was not reported. Later, Ciarka A. et al. investigated the impact of low-dose dopamine on weaning from mechanical ventilation in COPD patients [82]. Dopamine did not attenuate ventilation and had no effect on arterial blood gases. Effects of dopamine infusion on cardiovascular system were not reported.

#### Dobutamine

Dobutamine, a catecholamine derivative with specificity for beta-1 adrenergic receptors, is usually reserved for patients with severe reduction in cardiac output leading to compromised vital organ perfusion [39]. Theoretically, optimizing LV function with a short-acting inotropic agent seems to be a reasonable approach to weaning failure due to LV systolic dysfunction. However, the potential adverse effects of dobutamine, including increased heart rate with concomitant increased myocardial oxygen demand, render it unlikely to be beneficial. Furthermore, the sympathetic nervous system activation expressed by catecholamine excess during failed SBTs, does not support exogenous catecholamine administration. Finally, dobutamine is not indicated for treatment of cardiac failure with preserved ejection fraction (i.e., diastolic heart failure) [39, 47], which is a common cause of weaning failure of cardiovascular origin.

Indeed, when dobutamine at a dosage of 7 µg/kg/min was given in 10 difficult-to-wean COPD patients exhibiting weaning difficulties associated with increased PAOP during SBTs [83], the rate-pressure product which is a global index of myocardial workload and myocardial oxygen demand [84] was significantly increased. One could comment on the absence of a clear indication of dobutamine administration in those patients, since there was no evidence of impaired LV contractility, as shown by a LV ejection fraction within the normal range.

### Phosphodiesterase-3 inhibitors

Phosphodiesterase-3 inhibitors, such as milrinone, amrinone and enoximone, are another inotropic class of agents; they have attractive systemic and pulmonary vasodilatory properties potentially useful in right ventricular failure and pulmonary hypertension, but are often associated with hypotension and arrhythmias.

*Enoximone* has been evaluated in the treatment of LV dysfunction after cardiac surgery, in nine patients with one or more unsuccessful attempts of weaning due to LV dysfunction [20]. Enoximone was given at a dose of 10 µg/kg/min for the first 30 min followed by 30 µg/kg/min. During spontaneous ventilation, cardiac index increased by 34%, but mean arterial, right atrial pressure and PAOP did not change. Despite an increase in venous admixture due to augmented cardiac index and inhibition of hypoxic vasoconstriction, no oxygen debt occurred because of increased oxygen delivery. Seven of nine patients were weaned successfully from mechanical ventilation. Similarly, in six coronary patients with pulmonary edema after discontinuing mechanical ventilation, the administration of enoximone prevented LV dysfunction in five of them and enabled successful weaning [21]. No other studies exist; therefore, there are insufficient data to support enoximone use in clinical scenarios of weaning failure.

### Calcium sensitizer (Levosimendan)

Levosimendan is a novel calcium sensitizer and ATP-sensitive potassium channel opener. Its mode of action is completely independent of the β_1_-adrenergic pathway. In contrast to catecholamines, levosimendan enhances cardiac contractility without increasing myocardial oxygen demand and inducing negative impact on diastolic function [85]. In addition, levosimendan has a marked “decongestive” benefit; it reduces cardiac filling pressures and pulmonary vascular resistance through a vasodilator effect, decreasing, thus, RV afterload [86]. Therefore, it might be particularly beneficial for patients with concomitant pulmonary hypertension.

The clinical benefits of levosimendan in difficult-to-wean patients because of cardiovascular dysfunction have been highlighted in few reports [17, 83, 87–89]. Levosimendan was given in ventilator-dependent patients with impaired LV function, who had failed a SBT or extubation attempt, and were already under diuretic and vasodilator treatment. Following a 24-h infusion of levosimendan, weaning from mechanical ventilation was re-attempted. Levosimendan significantly improved LV ejection fraction and oxygenation variables and contributed to successful weaning in 7 out of 12 patients [17]. Also, the short-term hemodynamic effects of levosimendan, compared to dobutamine, were studied in 10 COPD patients experiencing weaning difficulties related to increased PAOP [83]. Levosimendan resulted in significantly greater inhibition of SBT-induced increase in PAOP and mean pulmonary artery pressure than dobutamine. Moreover, on mechanical ventilation, the rate-pressure product remained constant with levosimendan infusion, whereas it was significantly increased with dobutamine infusion. All included patients were ultimately extubated without the adjunct of any other cardiovascular drug. Lastly, use of levosimendan effectively contributed to successful weaning in difficult-to-wean patients with impaired LV function in a study available only in an abstract form [87] as well as in single patients with LV dysfunction of various etiologies [88, 89].

The therapeutic potential of levosimendan seems to be beyond its effects on LV performance [90]. As it has been shown experimentally in a canine model with compromised cardiac function, levosimendan improved the splanchnic mucosal oxygenation that was depressed by mechanical ventilation [91]. Furthermore, levosimendan improved the neuromechanical efficiency and contractile function of the human diaphragm in vivo (healthy subjects) as well as in vitro (muscle fibers from COPD patients’ diaphragm), suggesting a therapeutic approach to improve respiratory muscle function [92]. A currently ongoing randomized clinical trial (ClinicalTrials.gov identifier NCT01721434) has planned to determine whether levosimendan improves weaning outcome from mechanical ventilation. However, preexisting cardiovascular disease is an excluding factor in this protocol.

Taken together, levosimendan might exert a double beneficial effect during the critical period of weaning from mechanical ventilation in difficult-to-wean patients with impaired cardiovascular function. However, it should be noted that the addition of levosimendan to the standard treatment in adults with sepsis was associated with a lower likelihood of successful weaning from mechanical ventilation and a higher risk of supraventricular tachyarrhythmias [93]. Therefore, its efficacy and safety regarding this indication are still uncertain.

### Vasodilators

#### Nitroglycerin

**Fig. 4:**
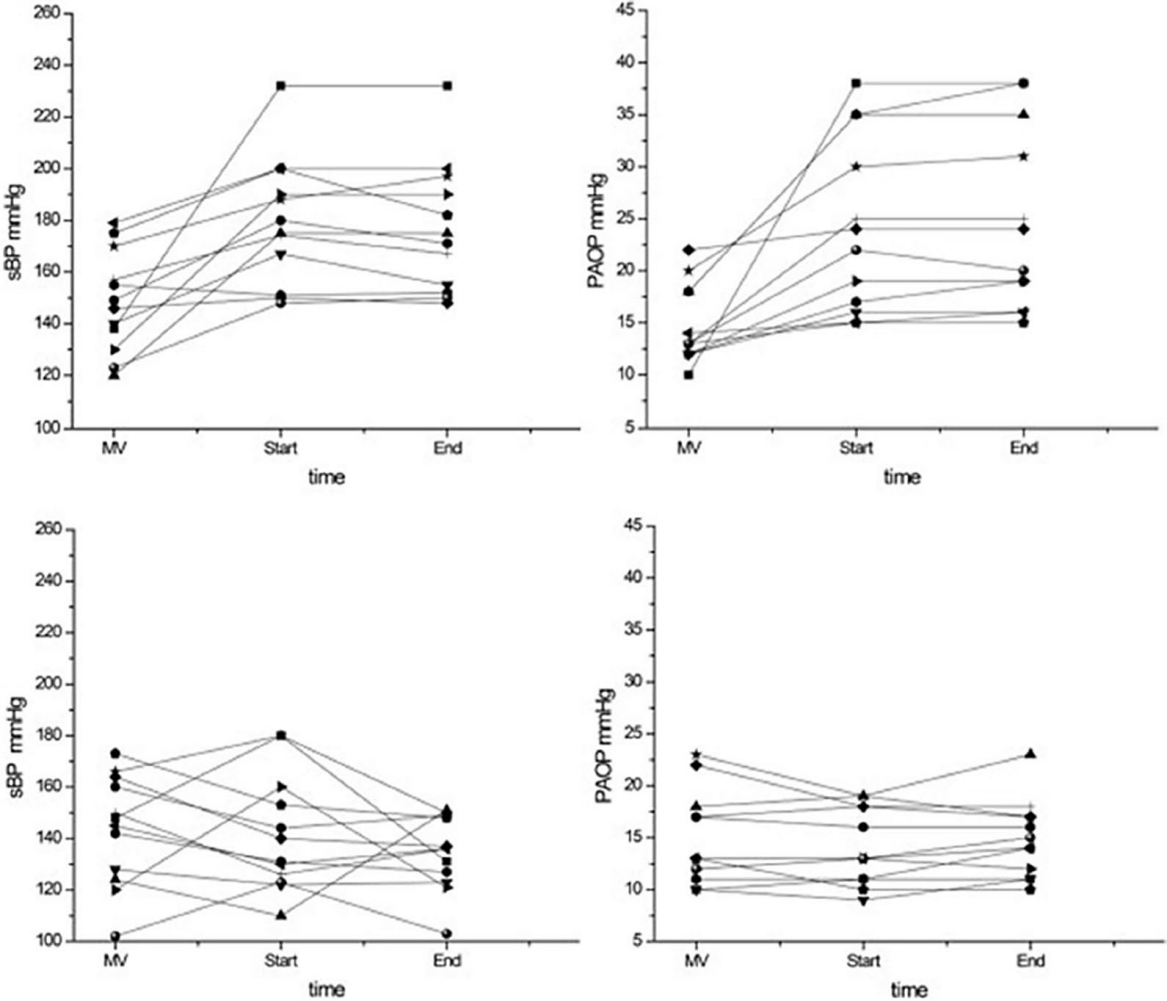
Individual values of systolic blood pressure (sBP) (left) and pulmonary artery occlusion pressure (PAOP) (right) obtained on mechanical ventilation (MV) and at the 10th minute (Start) and the last minute (End) of a spontaneous breathing trial without (upper panel) and with nitroglycerin treatment (lower panel) (adapted from [18])

By systemic venous and arterial vasodilatory properties, nitroglycerin results in reduction in venous return and LV afterload. In addition, by coronary vasodilation, it is beneficial in those patients with a concomitant coronary syndrome, either confirmed as such or highly suspected to develop. Nitroglycerine is a short-acting vasodilator having a rapid onset (2–5 min) and short duration of action (5–10 min). The hemodynamic and clinical effects of nitroglycerin were studied in COPD patients who repeatedly failed to wean from mechanical ventilation exhibiting systemic arterial hypertension [18]. While the mean systemic arterial pressure, rate-pressure product, mean pulmonary arterial pressure, and PAOP increased in failing trials, these variables remained stable under high dosing of nitroglycerin (Fig. 4). These favorable hemodynamic effects were associated with a successful SBT and extubation in 92% and 88% of patients, respectively. Interestingly, nitroglycerin treatment resulted in a lesser extent of arterial PO_2_ decrease and venous admixture increase compared to control, possibly indicating a reduction in weaning-induced acute pulmonary congestion. Other studies are lacking, thus, this treatment in weaning failure of cardiovascular origin remains to be further investigated.

### Phosphodiesterase-5 inhibitors (Sildenafil)

Phosphodiesterase-5 inhibitors have been used in selected mechanically ventilated patients with pulmonary hypertension and difficult weaning in order to take advantage of their short-term acute vasodilatory effects on pulmonary circulation. The first report included three COPD patients with repeated SBT failures [19]. These patients received 50 mg of sildenafil through the nasogastric tube under respiratory and hemodynamic monitoring with a pulmonary artery catheter. After sildenafil treatment, pulmonary artery pressure and PAOP decreased. The respiratory frequency to tidal volume index and the difference in arterial minus end-tidal carbon dioxide pressure decreased as well, indicating a beneficial effect of sildenafil on the pattern of breathing and reduction in the physiological dead space, respectively. The patients were successfully extubated. Similarly, sildenafil 20–30 mg three times daily enabled weaning from mechanical ventilation and inhaled nitric oxide in a patient with acute respiratory failure due to severe pulmonary hypertension and a right-to-left shunt through a patent foramen ovale [94]. Finally, in a mechanically ventilated patient with right ventricular dysfunction related to secondary pulmonary hypertension, sildenafil 25 mg every 8 h successfully substituted inhaled nitric oxide, inducing a sustained reduction in pulmonary pressures without any systemic adverse effect, in terms of systemic arterial pressure, cardiac index and mixed venous oxygen saturation, thus, facilitating the discontinuation of respiratory and cardiovascular organ support [95]. Phosphodiesterase-5 inhibition has also been reported to reverse bronchoconstriction [96] and to improve the pulmonary function [97]. However, it should be stressed that, sildenafil, despite its reported favorable results in pulmonary hypertension cases, cannot be currently recommended due to the limited evidence.

(4) Beta-blockers

Beta-blockers have occasionally been used to facilitate weaning in specific conditions. In one report of two elderly patients with weaning-induced pulmonary edema refractory to treatment with diuretics, vasodilators and inotropes, TTE data confirmed the diagnosis of hypertrophic obstructive cardiomyopathy and they were successfully managed with beta-blockers (atenolol 200 mg/day) and calcium channel blockers (diltiazem 300 mg/day) [98]. Similarly, beta-blockers in combination with angiotensin-converting enzyme inhibitors and mild negative water balance successfully treated cardiogenic pulmonary edema associated with LV diastolic dysfunction, unmasked during SBT [56], as well as in a trauma patient with documented coronary artery disease [99]. These reports highlight the need for an accurate diagnosis in order to individualize the management of weaning failure of cardiovascular origin.

### Non-pharmacological strategies

Beyond pharmacological treatments, other measures such as ventilatory strategies and/or cardiac interventional procedures, when indicated, have been used to improve the outcome of the weaning due to cardiovascular dysfunction.

1. Ventilatory strategies/SBT technique

The choice of the appropriate SBT technique is controversial. Cabello et al. compared SBT on T-piece with that on PSV, with and without PEEP, in patients who had failed a previous T-piece trial and had a pulmonary artery catheter in place [9]. During SBTs, patients on T-piece exhibited more negative intrathoracic pressures as well as greater increases in systemic blood pressure and PAOP, indicating that T-piece strategy might be more challenging for the heart compared to PSV with PEEP. Noteworthy, 50% of these patients had COPD and 80% of those who exhibited a PAOP above 18 mmHg during the failed T-piece trial had concomitant cardiovascular disease. Most patients succeeded the PSV trial, although all patients failed the T-piece one. A recent meta-analysis confirmed that PSV significantly reduces the work of breathing compared with T-piece [100]. Therefore, for patients with impaired cardiovascular function, gradual withdrawing of mechanical ventilation by PSV seems less stressful for the cardiovascular system than the abrupt stop by T-piece trial.

On the other hand, as it has been emphasized by Tobin [101], even a low level of PEEP during SBT through PSV can decrease the work of breathing by as much as 40% in ventilated patients and also can produce a substantial increase in cardiac output in patients with LV failure. Therefore, by applying “minimal ventilator settings,” i.e., PSV of 5 cm H_2_O with PEEP or continuous positive airway pressure (CPAP) of 5 cm H_2_O, causes physicians to overestimate the patient’s capacity to handle an increase in cardiorespiratory load following extubation, thus, resulting in extubation failure. Therefore, using a trial of unassisted breathing (i.e., T-piece trial) seems reasonable, especially in patients who might experience cardiorespiratory difficulties after extubation (i.e., those with impaired cardiovascular function), because this strategy may unmask the need for further optimization of cardiorespiratory function.

(2) Use of noninvasive ventilation in the post-extubation period as a weaning adjunct

Noninvasive ventilation (NIV) has been shown to be useful as adjunctive therapy in the treatment of respiratory failure in acute heart failure syndromes [102] such as acute cardiogenic pulmonary edema, including cases due to LV diastolic dysfunction [103]. Also, NIV has an important role in the treatment or prevention of post-extubation respiratory failure in selected high-risk patient groups [104–106]. Recent guidelines issued by the American Thoracic Society and the American College of Chest Physicians recommend, for patients at high risk of extubation failure (elderly, COPD, congestive heart failure, or hypercapnia during the SBT) who have been receiving mechanical ventilation for > 24 h and passed a SBT, extubation to preventive NIV as superior to nonpreventive NIV regarding extubation success, ICU, and both short- and long-term mortality [107]. Physicians who choose to use NIV should apply such treatment immediately after extubation to realize the outcome benefits.

Finally, high-flow nasal cannula oxygen therapy, an evidence-based modality for treatment of hypoxemic respiratory failure, was found noninferior to NIV in post-extubation settings for patients at high risk of respiratory failure [108]. However, there is no data regarding its use after extubation in patients at high risk of weaning-induced pulmonary edema.

(3) Cardiac interventional procedures

Medical treatment of weaning failure due to cardiovascular dysfunction may be ineffective in certain circumstances, such as in critically ill patients with myocardial ischemia or/and in the setting of valvulopathy. In such cases, further therapeutic approaches should be considered.

Persistent weaning failure from mechanical ventilation because of pulmonary edema due to myocardial ischemia has been successfully treated with coronary angioplasty in two patients with known preexisting myocardial ischemia [109, 110]. The intervention was followed by improvement in coronary perfusion that allowed successful extubation. Similarly, in another case in which weaning was impossible, percutaneous balloon mitral commissurotomy for severe mitral stenosis was successfully performed, resulting in removal of the ventilator 24 h later [30]. In another patient with COPD and extensive myocardial ischemia, previously unknown, detected after repeated failing SBTs, a coronary artery bypass graft surgery was performed because optimal pharmaceutical treatment proved ineffective and coronary angioplasty was not technically feasible due to extensive stenotic lesions [111]. The patient was successfully weaned the third postoperative day. Finally, cardiac resynchronization therapy may assist weaning from circulatory and respiratory support in critically ill patients with LV systolic dysfunction [112].

## Conclusion

Cardiovascular dysfunction is an important cause or contributor to weaning failure. Early identification of high-risk patients for weaning failure of cardiovascular origin and accurate diagnosis is crucial, as targeted treatment according to the underlying mechanism might help the heart to tolerate more effectively the burden of weaning process.

Specific evidence-based recommendations for treating confirmed weaning failure of cardiovascular origin cannot be applied, at least at the moment, due to the lack of data derived from clinical trials. However, based on both pathophysiology and data derived from small-scale studies, causal pharmacological approach is being applied in real life daily practice, such as prompt diuresis in excessive preload, nitrates in excessive afterload and/or myocardial ischemia or other rational treatment in specific indications. Until appropriate evidence emerges, tailoring cardiovascular treatment and monitoring the individual responses to therapy should be carefully performed by ICU clinicians, reminding that much further research is required.

## Abbreviations

ICU: intensive care unit
PSV: pressure support ventilation
COPD: chronic obstructive pulmonary disease
SBT: spontaneous breathing trial
SvO_2_: mixed venous oxygen saturation
ScvO_2_: central venous oxygen saturation
TTE: transthoracic echocardiography
TDI: tissue Doppler imaging
TP: transpulmonary
PA: pulmonary artery
PAOP: pulmonary artery occlusion pressure
LV: left ventricular
BNP: B-type natriuretic peptide
EVLW: extravascular lung water
PLR: passive leg raising
ACE: angiotensin-converting enzyme
CPAP: continuous positive airway pressure
NIV: noninvasive ventilation
